# Diagnostic utility of neurogenic biomarkers in differentiating sepsis with and without associated encephalopathy: a systematic review and meta-analytic approach

**DOI:** 10.3389/fneur.2025.1640618

**Published:** 2025-10-28

**Authors:** Xiaofei Lin, Jun Zhang, Tailiang Ren, Haixia Cao, Cheng Chang, Yumei Wang

**Affiliations:** ^1^Department of Pediatrics, Huai'an Maternal and Child Health Care Hospital Affiliated to Yangzhou University and the Huai'an Maternity and Child Clinical College of Xuzhou Medical University, Huai'an, Jiangsu, China; ^2^Department of Pediatrics, The Affiliated Hospital of Qinghai University, Xining, Qinghai, China; ^3^Department of Neurology, Affiliated Hospital of Nanjing University of Chinese Medicine, Nanjing, Jiangsu, China; ^4^Neonatal Disease Screening Center, Huai'an Maternal and Child Health Care Hospital Affiliated to Yangzhou University, The Huai'an Maternity and Child Clinical College of Xuzhou Medical University, Huai'an, Jiangsu, China

**Keywords:** sepsis-associated encephalopathy, NSE, biomarker, S100β, meta-analysis, systematic review

## Abstract

**Background:**

Sepsis-associated encephalopathy (SAE) is a frequent complication of sepsis, manifesting as acute brain dysfunction and often resulting in persistent cognitive deficits, neurological impairment, and increased mortality. Timely and accurate diagnosis of SAE is essential to guide therapeutic decisions and improve clinical outcomes. In recent years, neurogenic biomarkers have emerged as potential serum-based indicators for the diagnosis and progression monitoring of SAE.

**Methods:**

A comprehensive search of PubMed/MEDLINE, Embase, the Cochrane Library, Web of Science, and Scopus was conducted from inception to 30 April 2025. Weighted mean differences (WMDs) and 95% confidence intervals (CIs) were calculated using a random-effects model.

**Results:**

Forty-seven studies (50 arms) were included. Random-effects analysis revealed significant differences in serum NSE levels between SAE and NE adult patients (WMD = 6.82; 95% CI: 5.43, 8.21; *P* < 0.001), S100β levels (WMD = 0.48; 95% CI: 0.37, 0.60; *P* < 0.001), GFAP levels in the SAE group (WMD = 62.28; 95% CI: 45.42, 79.14; *P* < 0.001), TAU levels in the SAE individuals (WMD = 1.73; 95% CI: 0.95, 2.51; *P* < 0.001), UCH-L1 levels in SAE patients (WMD = 1.73; 95% CI: 0.95, 2.51; *P* < 0.001), APACHE II scores in the SAE group (WMD = 6.30; 95% CI: 4.61, 7.99; *P* < 0.001), and SOFA scores in SAE (WMD = 3.65; 95% CI: 2.96, 4.34; *P* < 0.001).

**Conclusion:**

Elevated serum levels of neurogenic biomarkers may serve as potential predictors of SAE and are associated with increased mortality in septic patients. These biomarkers show promise as reliable, minimally invasive tools for diagnosis and longitudinal monitoring of SAE. However, these findings should be interpreted with caution due to substantial heterogeneity across the included studies.

## Introduction

Sepsis-associated encephalopathy (SAE) is a common and severe complication of sepsis, manifesting as diffuse cerebral dysfunction without overt central nervous system infection. The reported incidence of SAE varies widely from 9% to over 70% of septic patients, depending on diagnostic criteria and patient populations, with some studies suggesting rates as high as 83.6% in intensive care settings (1, 2). Clinically, SAE ranges from subtle delirium to deep coma and is independently associated with prolonged hospitalization, long-term cognitive impairment, and increased mortality (3).

The pathogenesis of SAE is multifactorial. Systemic inflammation disrupts the blood–brain barrier, allowing cytokines and immune cells to infiltrate the brain; concomitant microcirculatory dysfunction and metabolic derangements lead to hypoxia, oxidative stress, and neurogenic injury. Activated microglia and astrocytes further propagate neuroinflammation, while neurotransmitter imbalances contribute to encephalopathy. Although these mechanisms are well characterized, their relative contributions vary among individuals, complicating early and accurate diagnosis (4, 5).

Given the limitations of clinical evaluation and neuroimaging in diagnosing SAE, there has been growing interest in circulating neurogenic biomarkers as objective indicators of CNS injury. Key candidates include neuron-specific enolase (NSE), S100 calcium-binding protein B (S100β), glial fibrillary acidic protein (GFAP), tau protein (TAU), and ubiquitin C-terminal hydrolase L1 (UCH-L1) (6, 7). NSE and S100β originate predominantly from neurons and astrocytes, respectively; elevated serum levels have been linked to neurogenic damage and correlate with delirium severity (8). GFAP reflects astroglial injury, while TAU and UCH-L1 indicate microtubule disruption and ubiquitin-proteasome pathway alterations (9, 10). Early studies demonstrated significant increases in these biomarkers among SAE patients compared to septic patients without encephalopathy (NE), suggesting their potential utility in both diagnosis and prognostication (11–13).

Previous meta-analyses have demonstrated that elevated NSE levels are significantly associated with SAE and poorer outcomes (14), while higher S100β concentrations correlate moderately with SAE incidence and mortality risk (15). However, these reviews focused on single biomarkers and often exhibited high between-study heterogeneity. Evidence for GFAP, TAU, and UCH-L1 remains limited to small cohorts, and few studies have evaluated all five markers in parallel or examined their comparative diagnostic performance (2, 16). Moreover, the influence of covariates such as age, sepsis severity, sampling time, and assay variability on biomarker levels has not been systematically assessed.

To address these gaps, our study aims to (1) quantify the differences in serum levels of NSE, S100β, GFAP, TAU, and UCH-L1 between SAE and NE patients through pooled effect estimates; (2) evaluate diagnostic accuracy using subgroup and meta-regression analyses based on age, timing of sampling, and sample size; and (3) explore associations between biomarker concentrations and clinical outcomes, including organ dysfunction scores and mortality. By integrating data across multiple neurogenic biomarkers, this meta-analysis will clarify their relative and combined utility for the early identification and risk stratification of SAE, ultimately informing clinical decision-making and guiding future research.

## Methods

### Search strategy and selection criteria

This systematic review and meta-analysis was conducted in accordance with the Preferred Reporting Items for Systematic Reviews and Meta-Analyses (PRISMA) guidelines (17). Two investigators independently searched PubMed/MEDLINE, Embase, the Cochrane Library, Web of Science, and Scopus from database inception through April 30, 2025. The search combined terms related to “sepsis,” “encephalopathy,” and each neurogenic biomarker of interest (neuron-specific enolase, S100β, glial fibrillary acidic protein, tau protein, ubiquitin C-terminal hydrolase L1), using controlled vocabulary (e.g., MeSH and Emtree) and free-text keywords. No language restrictions were applied. Reference lists of included studies and relevant reviews were manually screened to identify additional eligible reports.

### Study selection

Titles and abstracts retrieved through electronic searches were independently screened by two reviewers for relevance to adult septic patients with and without encephalopathy. Full texts were obtained for all studies deemed potentially eligible. We included observational cohort and case–control studies that reported quantitative serum or plasma levels of at least one of the specified neurogenic biomarkers in both SAE and non-encephalopathic sepsis (NE) groups. Studies were excluded if they lacked a comparator group without encephalopathy, did not report sufficient data to calculate mean differences and standard deviations, involved pediatric populations exclusively, or were case reports, reviews, conference abstracts, or animal studies. Disagreements were resolved through discussion or consultation with a third reviewer.

### Data extraction

A standardized data collection form was used to extract characteristics from each eligible study, including first author, publication year, country, study design, patient demographics (mean age, gender distribution), sepsis definitions, criteria for encephalopathy, timing of biomarker sampling relative to sepsis onset, assay methods, and sample size per group. Outcome data comprised mean (and standard deviation) or median (and interquartile range) biomarker concentrations for SAE and NE groups, along with clinical outcomes such as APACHE II and SOFA scores and mortality rates. When necessary, medians and interquartile ranges were converted to means and standard deviations using established formulas.

### Quality assessment

Study quality and risk of bias were independently appraised by two reviewers using the Cochrane-endorsed Quality Assessment of Diagnostic Accuracy Studies version 2 (QUADAS-2) tool (18). This framework evaluates four key domains—patient selection, conduct and interpretation of the index test, reference standard, and flow and timing—to assign judgments on bias risk and applicability. Any discrepancies between reviewers were discussed and reconciled to reach a consensus.

### Data synthesis and statistical analysis

Meta-analyses were conducted using a random-effects model (DerSimonian and Laird) to account for between-study variability (19). Weighted mean differences (WMDs) with corresponding 95% confidence intervals (CIs) were calculated for each biomarker comparing the SAE to the NE groups. Heterogeneity was quantified using the *I*^2^ statistic, with values above 50% indicating substantial heterogeneity, and tested for significance using Cochran's Q. To explore the sources of heterogeneity, subgroup analyses were planned a priori by patient age, timing of sample collection, and study sample size. Meta-regression was performed to evaluate the impact of continuous covariates, including mean APACHE II score, assay type (e.g., ELISA vs. automated analyzer), and publication year, on effect estimates when at least 10 studies were available.

Potential publication bias was assessed through visual inspection of funnel plots and quantitatively tested with Egger's regression (20) and Begg's rank correlation tests (21). Duval and Tweedie's trim-and-fill method was applied to adjust the pooled estimates if asymmetry suggested missing studies (22). Sensitivity analyses were conducted by sequentially omitting each study (leave-one-out) to evaluate the influence of individual studies on overall results. All statistical analyses were performed using Stata version 17, with two-sided *P*-values of <0.05 considered statistically significant.

## Results

### Study characteristics

**Figure 1:**
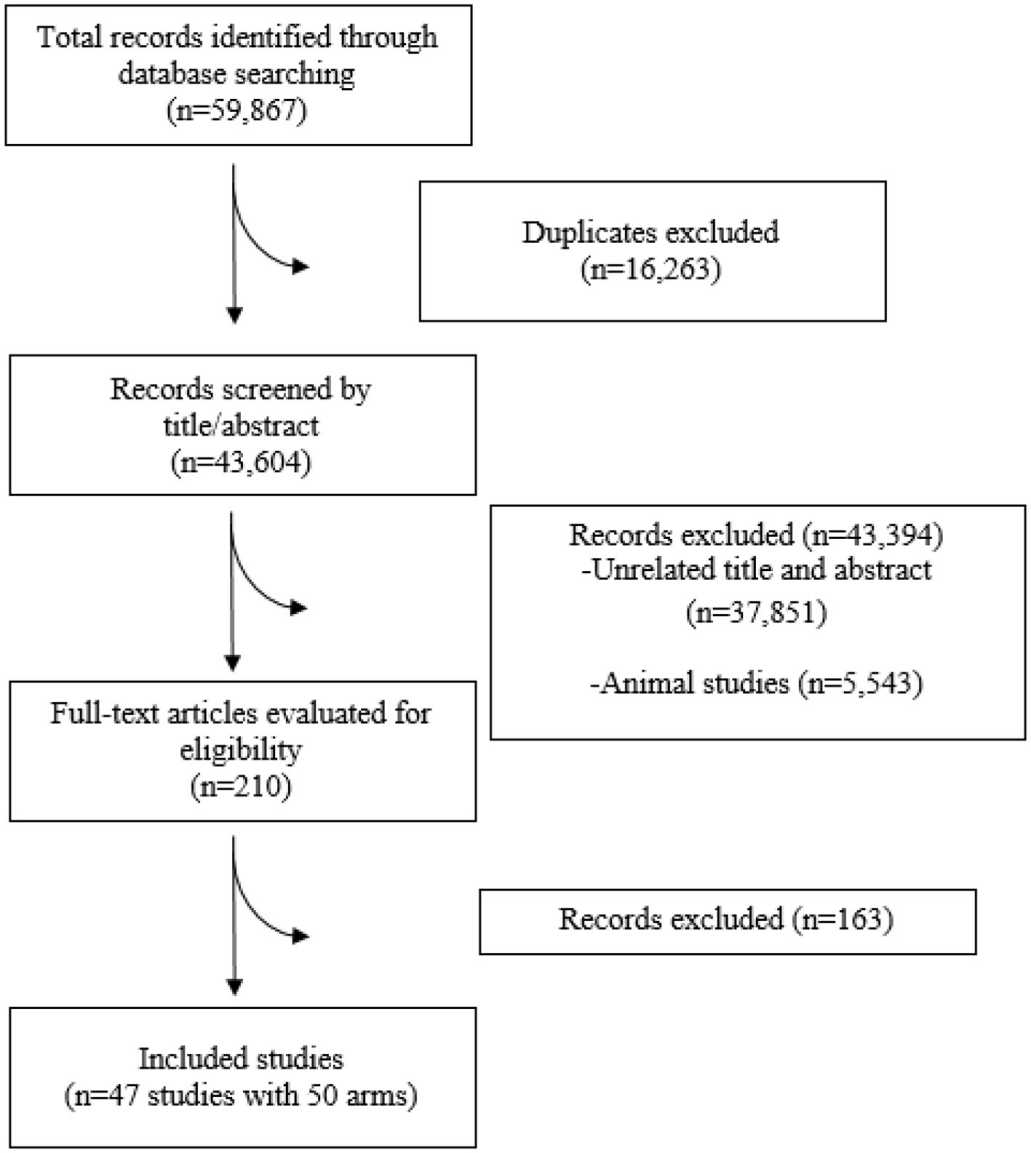
Study selection flow diagram.

Figure 1 presents the PRISMA flowchart detailing the study selection process. The electronic databases search yielded 59,867 articles, of which 16,263 articles were detected as duplicates. Accordingly, 43,604 studies underwent a screening process based on titles and abstracts, leading to 210 articles retained for full-text evaluation. Eventually, 47 studies (50 arms) matched our inclusion criteria and were included in the meta-analysis. Study characteristics of all included studies are provided in Table 1. The included studies were conducted from 2000 to 2024, with mean ages of adult participants ranging from 27 to 80 years. Both genders (men and women) were included. Samples were collected over periods ranging from 1 to 3 days.

**Table 1:** Summary of included studies.

**Author**	**Year**	**N total**	**Sex**	**Age SAE (year)**	**Age NE (year)**	**Sample collection time (day)**
Lin et al.	2024	224	Both	44.45	-	1, 2
Tan et al.	2024	177	Both	71.55	-	-
Cao et al.	2023	100	Both	58.51	57.69	Day of the medical visit
Chen et al.	2023	90	Both	64.3	-	-
Cui et al.	2022	200	Both	72.78	72.86	Within 48 h
Li et al.	2022	41	Both	37	38	12, 24, 48 h
Wang et al.	2022	80	Both	55.42	56.37	1, 3 day
Xiao et al.	2022	149	Both	42.78	40.26	ICU admission
Yu et al.	2022	162	Both	70.3	69.7	NR
Zhao1 et al.	2022	60	Both	55.89	55.23	NR
Zhao-2 et al.	2022	163	Both	NR	NR	ICU admission
Zhu et al.	2022	186	Both	55.45	55.48	Within 48 h
de Araujo et al.	2022	27	Both	3–6 months	-	-
Cui et al.	2022	200	Both	72.78	72.86	Within 48 h
Li et al.	2022	41	Both	37	38	12, 24, 48 h
Wang et al.	2022	80	Both	55.42	56.37	1, 3 day
Yu et al.	2022	162	Both	70.3	69.7	NR
Zhao et al.	2022	60	Both	55.89	55.23	NR
Kang et al.	2022	47	Both	27.5	21	Within 24 h
Li et al.	2022	41	Both	37	-	1, 2
Li-2 et al.	2022	72	Both	58.29	-	1
Yang et al.	2022	88	NR	80	-	NR
Zhang et al.	2022	75	Both	75.72	71.46	1, 4 day
Guo et al.	2021	120	Both	57.61	56.91	NR
Nong et al.	2021	96	Both	8.68	-	-
Jiang et al.	2021	64	Both	42.45	41.2	4 h
Chen et al.	2020	42	Both	68	58	ICU admission
Meng et al.	2020	178	Both	59.54	60.32	NR
Yuan-1 et al.	2020	184	Both	58.6	56.7	NR
Hui et al.	2020	60	Both	50.5	50.8	24 h after admission
Yuan (NE) et al.	2020	56	Both	-	56.07	NR
Yuan (SAE) et al.	2020	128	Both	58.6	-	NR
Zhou et al.	2019	38	Both	53	46	1 day
Yan et al.	2019	58	Both	55.8	55.0	Within 24 h
Wu et al.	2019	58	Both	NR	NR	Within 24 h
Orthun et al.	2019	86	Both	53.2	-	The first few hours
El Shimy et al.	2018	96	Both	Neonates	Neonates	After birth follow up
Erikson et al.	2019	22	Both	62.4	61.8	When CAM-ICU assessed
Kristo et al.	2018	22	Both	64.2	-	-
Liao et al.	2017	38	Both	55	51	1, 3 day
Feng et al.	2017	59	Both	52	57	1, 3 day
Lu et al.	2016	86	Both	59	58	NR
Nguyen et al.	2014	128	NR	65	-	ICU admission, 4 day
Yao et al.	2014	112	Both	56	52	1 day
Zhan et al.	2013	34	Both	57	-	1 h
Zhang et al.	2012	232	Both	51.5	-	-
Lin et al.	2012	50	Both	51	51	-
Li et al.	2011	50	Both	52	48	-
Hamed et al.	2009	40	NR	51.75	-	NR
Weigand et al.	2000	29	NR	-	-	1 day

### Methodological quality

**Figure 2:**
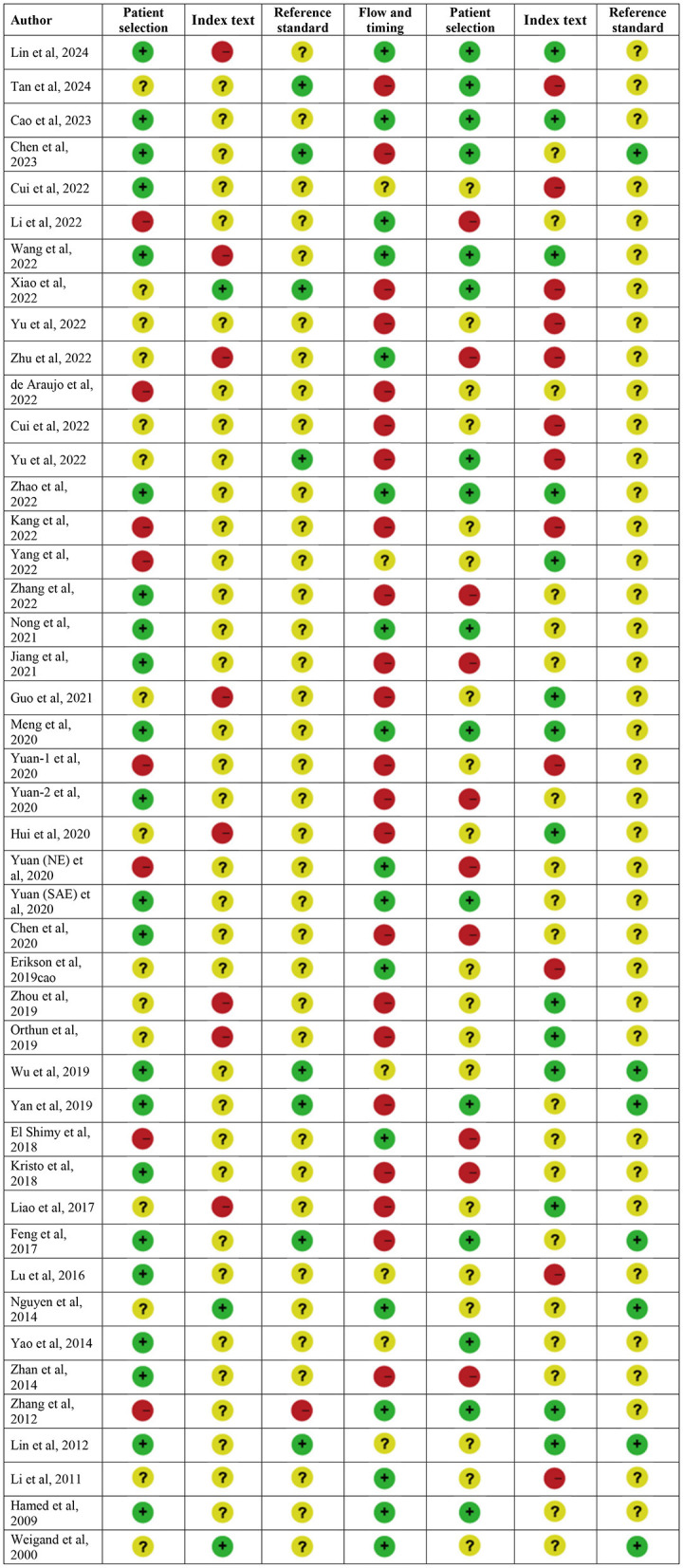
Quality assessment.

Quality assessment was conducted using the QUADAS-2 tool and is presented in Figure 2. The quality of the included studies varied. Overall, concerns regarding the applicability of the included studies to the review question were less significant than our concerns about the risk of bias. High risk of bias was mainly focused on flow and timing, and high applicability concerns mostly came from patient selection and index text, which may be attributed to various diagnostic criteria of SAE.

### Meta-analysis results

#### NSE in adults

**Figure 3:**
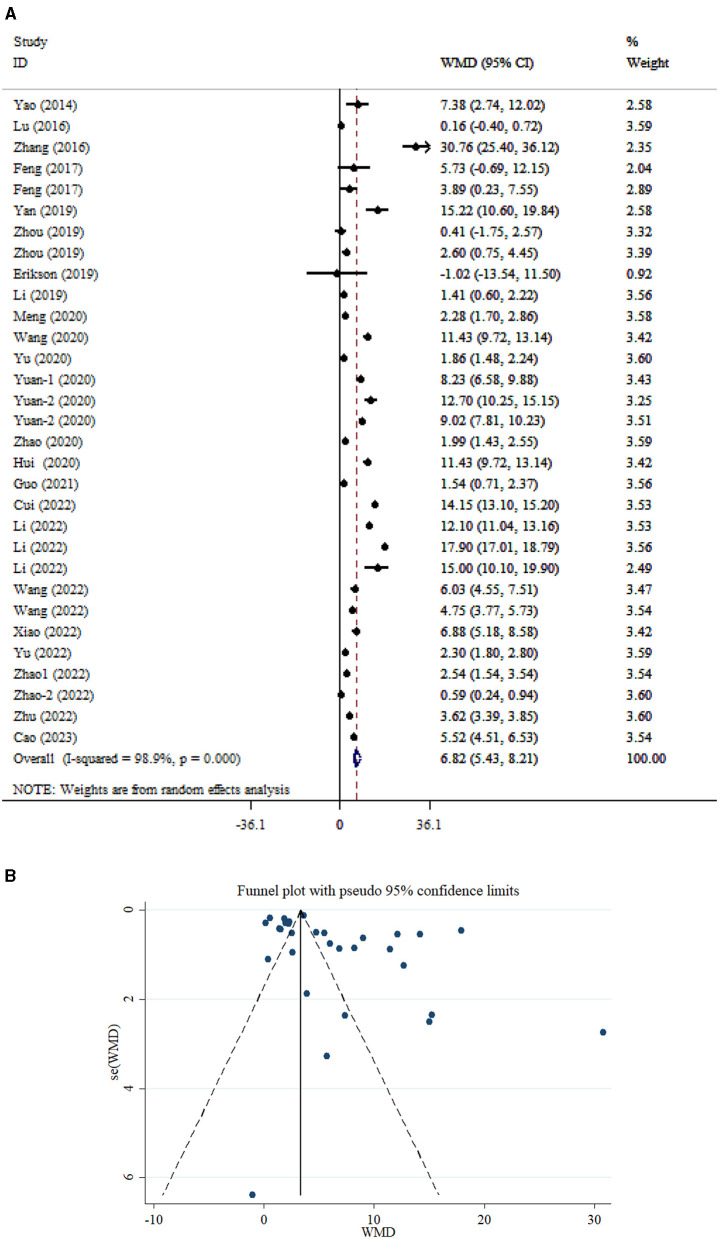
Forest plot **(A)** and funnel plot **(B)** evaluate the association between serum neurogenic biomarker levels and NSE.

A total of 31 studies (23–42) encompassing 3,216 participants were included in the analysis comparing serum NSE levels between patients with SAE and those without encephalopathy (NE). Random-effects analysis revealed a significant difference in serum NSE levels between SAE and NE adult patients (WMD = 6.82; 95% CI: 5.43, 8.21; *P* < 0.001; *I*^2^ = 98.9%, *P* < 0.001; Figure 3A). Subgroup analysis based on age demonstrated that serum NSE level in both SAE and NE adults was significantly elevated in both younger (<55 years) and older (>55 years) individuals (*P* < 0.05; Table 2). Moreover, subgroup analysis based on timing of sample collection showed that NSE levels were significantly higher in patients with SAE compared to those without NE, both when samples were collected within 1 day (*P* < 0.001) and after 1 day (*P* < 0.001) of sepsis onset. This finding indicates a significant elevation of NSE in SAE patients regardless of sampling time. In addition, serum NSE levels were significantly elevated in studies with sample sizes both below 100 and those with 100 or more participants. A small-study effect was observed in Egger's and Begg's tests (*P* = 0.020 and 0.012, respectively). However, visual inspection of the funnel plot (Figure 3B) revealed asymmetric distribution.

**Table 2:** Subgroup analyses for the comparison between outcomes.

**Subgroups**	**NO**	**SMD (95% CI)**	**P-within**	***I*^2^ (%)**	**P-heterogeneity**
**NSE**					
**Age SAE patients (year)**					
≤ 50	11	8.12 (3.47, 12.76)	0.001	99.3	<0.001
>50	18	6.51 (5.07, 7.95)	<0.001	98.2	<0.001
NR	2	0.91 (0.13, 1.70)	0.023	69.9	0.068
**Age NE patients (year)**					
≤ 50	12	9.03 (4.86, 13.20)	<0.001	99.2	<0.001
>50	17	5.71 (4.29, 7.13)	<0.001	98.0	<0.001
NR	2	0.91 (0.13, 1.70)	0.023	69.9	0.068
**Sample collection time (day)**					
≤ 1	12	9.41 (6.18, 12.64)	<0.001	98.3	<0.001
>1	8	8.82 (4.14, 13.51)	<0.001	99.5	<0.001
NR	11	2.96 (1.96, 3.95)	<0.001	95.7	<0.001
**Sample size**					
≤ 100	17	8.66 (5.28, 12.03)	<0.001	99.0	<0.001
>100	14	4.67 (3.39, 5.95)	<0.001	98.4	<0.001
***S100**β*					
**Age SAE patients (year)**					
≤ 50	12	0.74 (0.42, 1.05)	<0.001	99.9	<0.001
>50	17	0.36 (0.27, 0.45)	<0.001	97.4	<0.001
NR	4	0.20 (0.03, 0.36)	0.023	97.2	<0.001
**Age NE patients (year)**					
≤ 50		0.90 (0.53, 1.27)	<0.001	99.9	<0.001
>50		0.30 (0.21, 0.38)	<0.001	96.9	<0.001
NR		0.18 (0.08, 0.27)	<0.001	96.6	<0.001
**Sample collection time (day)**					
≤ 1	15	0.51 (0.37, 0.66)	<0.001	99.3	<0.001
>1	10	0.64 (0.30, 0.98)	<0.001	99.9	<0.001
NR	8	0.23 (0.15, 0.31)	<0.001	93.1	<0.001
**Sample size**					
≤ 100	20	0.65 (0.48, 0.83)	<0.001	99.8	<0.001
>100	13	0.22 (0.16, 0.27)	<0.001	94.2	<0.001
**TAU**					
**Sample collection time (day)**			0.020		
≤ 1	4	1.66 (0.26, 3.06)	<0.001	97.3	<0.001
>1	6	1.78 (0.96, 2.60)		90.2	<0.001
**Mortality**					
**Age SAE patients (year)**					
≤ 50	3	−0.09 (−3.97, 3.79)	0.963	98.4	<0.001
>50	12	−3.61 (−5.96, −1.25)	0.003	95.1	<0.001
NR	2	−5.72 (−9.55, −1.90)	0.003	71.6	0.060
≤ 50	3	4.11 (−2.64, 10.86)	0.233	96.2	<0.001
>50	5	−0.25 (−3.07, 2.58)	0.865	96.5	<0.001
NR	9	−7.96 (−10.50, −5.42)	<0.001	89.0	<0.001
**Sample collection time (day)**					
≤ 1	8	−2.02 (−4.21, 0.17)	0.070	94.8	<0.001
>1	2	−18.63 (−22.19, −15.06)	<0.001	0.0	0.512
NR	7	−1.87 (−4.62, 0.88)	0.182	96.2	<0.001
**Sample size**					
≤ 100	11	−5.88 (−7.36, −4.40)	<0.001	94.3	<0.001
>100	6	4.27 (−2.91, 11.45)	0.243	96.6	<0.001

#### NSE in children

**Figure 4:**
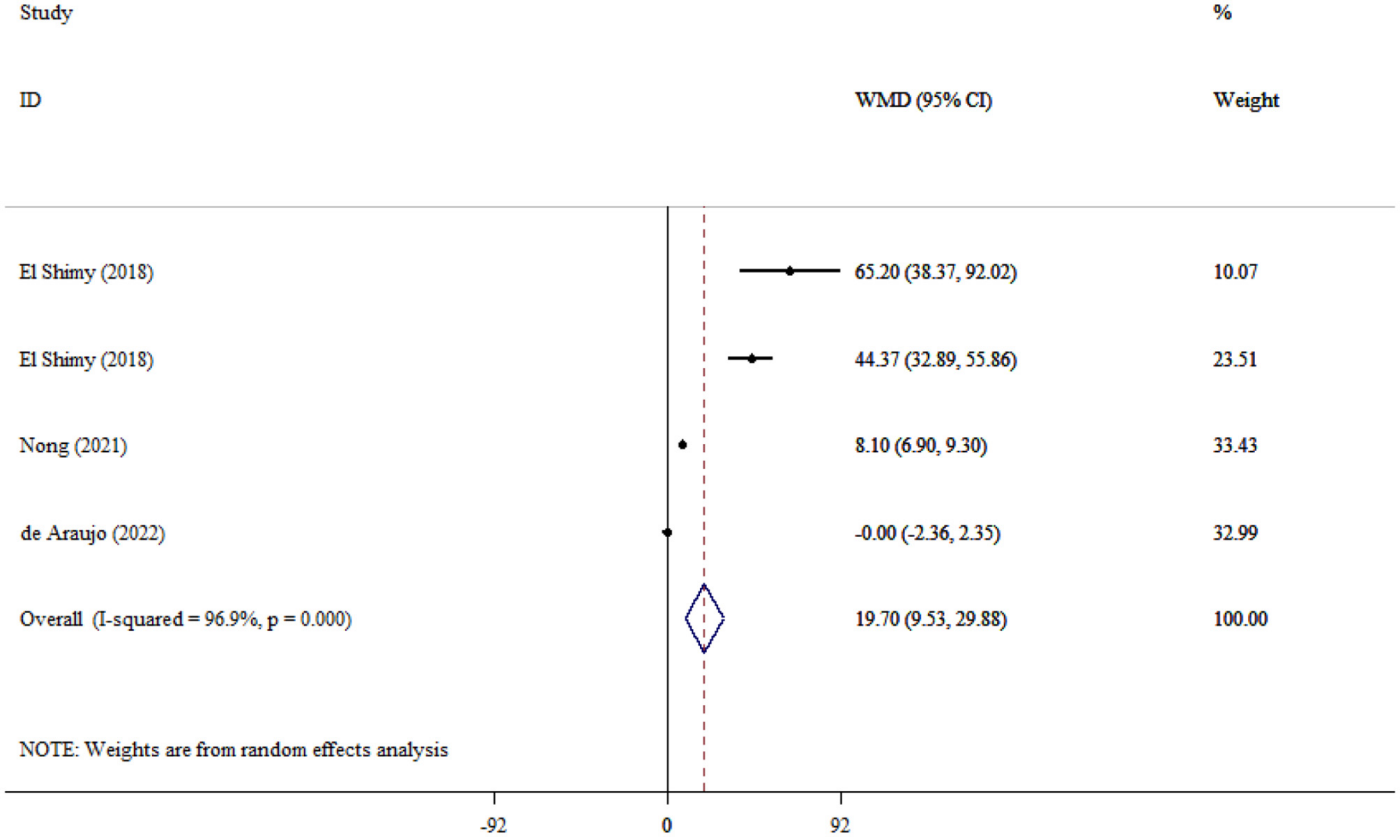
Forest plot evaluates the association between serum neurogenic biomarker levels and NSE in children.

A total of four studies encompassing 315 children showed a significant difference in serum NSE level between SAE and NE children (WMD = 19.70; 95% CI: 9.53, 29.88; *P* < 0.001; *I*^2^ = 96.9%, *P* < 0.001; Figure 4).

#### S100β

**Figure 5:**
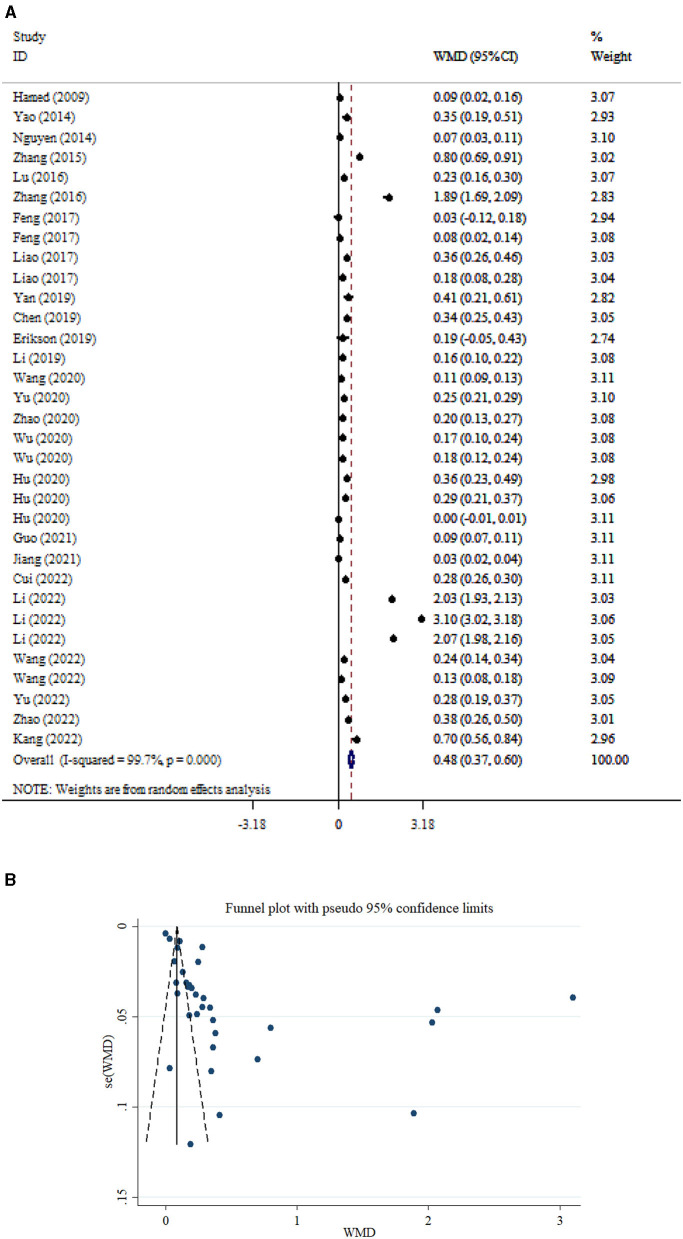
Forest plot **(A)** and funnel plot **(B)** evaluate the association between serum neurogenic biomarker levels and S100β.

A total of 33 studies encompassing 2,819 participants were included in the analysis comparing S100β levels between patients with SAE and NE patients. The pooled analysis demonstrated that S100β levels were significantly higher in SAE patients compared to NE patients (WMD = 0.48; 95% CI: 0.37, 0.60; *P* < 0.001; *I*^2^ = 99.7%, *P* < 0.001; Figure 5A). In addition, subgroup analysis indicated that S100β levels were significantly elevated in both younger (<55 years) and older (>55 years) adults, regardless of whether they had SAE or NE (*P* < 0.001; Table 2). Moreover, S100β levels were significantly higher in SAE patients compared to those without NE, both when samples were collected within 1 day (*P* < 0.001) and after 1 day (*P* < 0.001) of sepsis onset. In addition, serum S100β levels were significantly elevated in studies with sample sizes both below 100 and those with 100 or more participants. A significant small-study effect was observed in Egger's and Begg's tests (*P* = 0.034 and 0.001, respectively). However, visual inspection of the funnel plot (Figure 5B) revealed an asymmetric distribution.

#### GFAP

**Figure 6:**
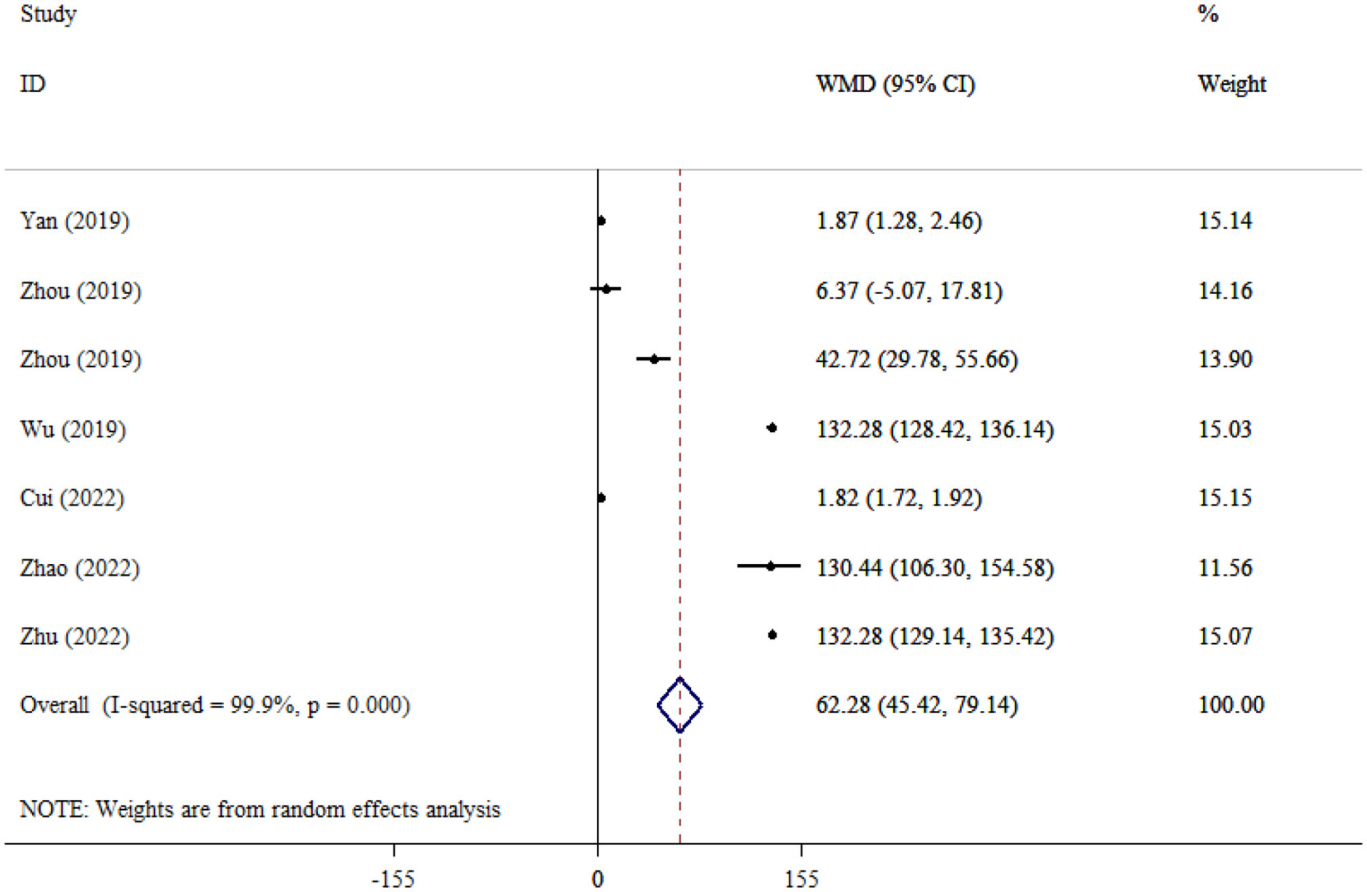
Forest plot evaluating the association between serum neurogenic biomarker levels and GFAP.

Seven studies (16, 27, 28, 37, 41, 42), including 871 patients, compared GFAP levels between SAE and NE patients and found that GFAP levels were significantly higher in the SAE group (WMD = 62.28; 95% CI: 45.42, 79.14; *P* < 0.001), indicating pronounced astroglial injury. Considerable heterogeneity was observed across studies (*I*^2^ = 99.9%, *P* < 0.001; Figure 6).

#### TAU

**Figure 7:**
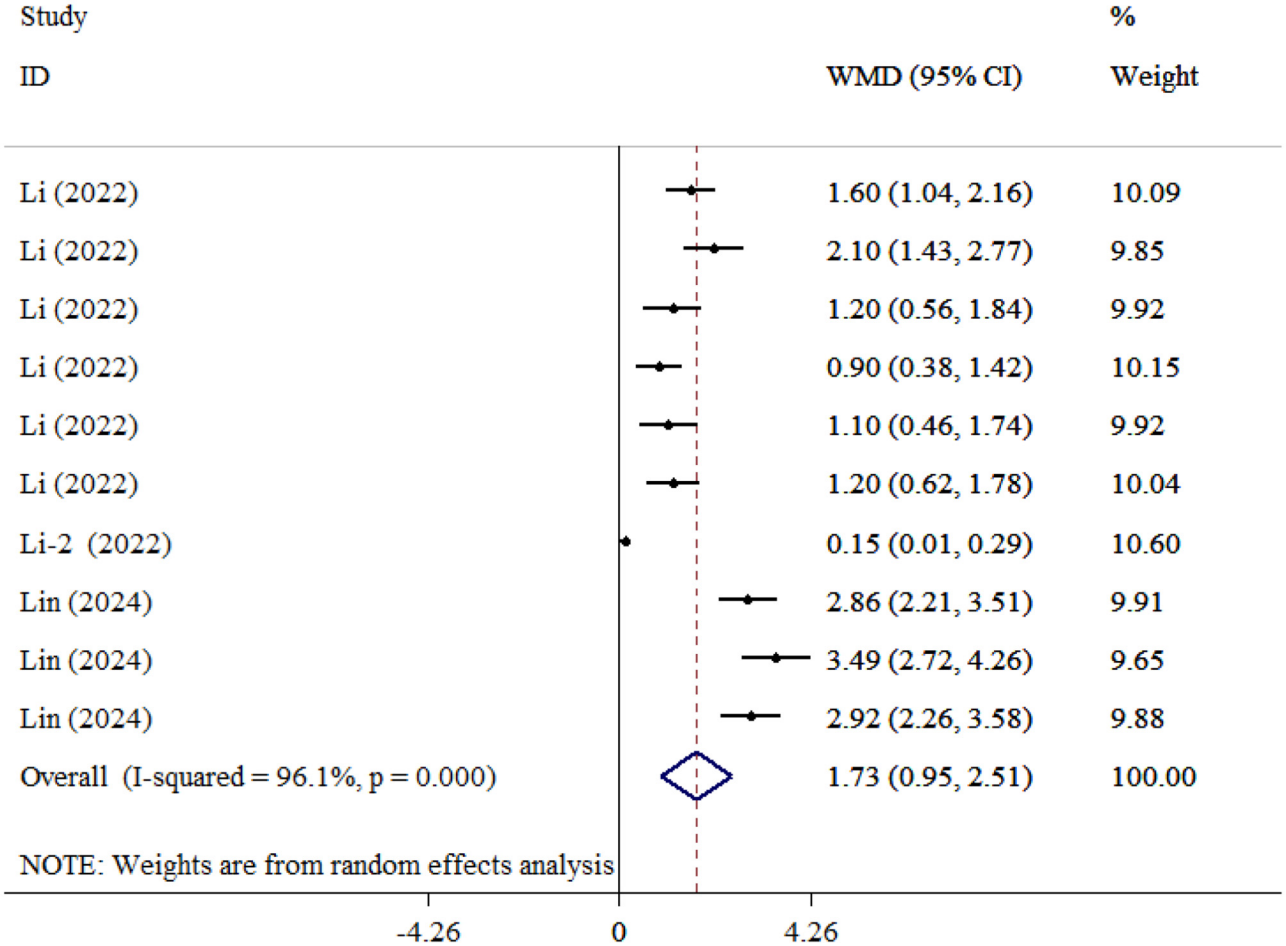
Forest plot evaluates the association between serum neurogenic biomarker levels and TAU.

A total of 10 studies (990 participants) (38, 43), comparing Tau protein levels between patients with SAE and NE patients, were included in the analysis. The combined effect analysis elucidated that TAU levels were significantly higher in SAE individuals (WMD = 1.73; 95% CI: 0.95, 2.51; *P* < 0.001; *I*^2^ = 96.1%, *P* < 0.001; Figure 7). Furthermore, subgroup analysis based on time of sampling indicated that TAU levels were significantly elevated in SAE patients compared to those with NE, both when samples were collected within 1 day (*P* < 0.001) and after 1 day (*P* < 0.001) of sepsis onset (Table 2).

#### UCH-L1

**Figure 8:**
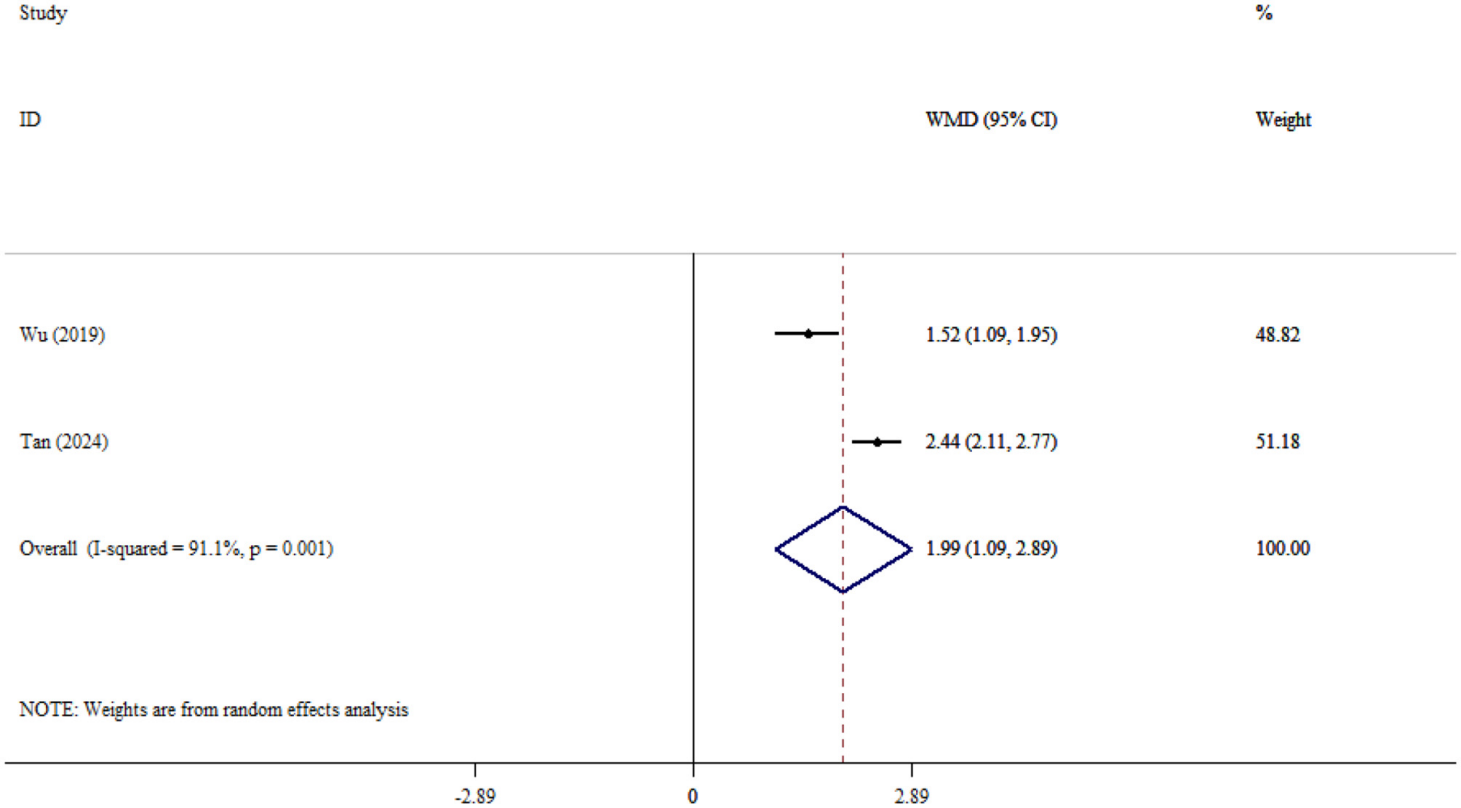
Forest plot evaluates the association between serum neurogenic biomarker levels and UCH-L1.

Two studies evaluated UCH-L1 levels between SAE and NE patients, and the pooled analysis showed elevated levels of UCH-L1 in SAE compared to NE patients (WMD = 1.73; 95% CI: 0.95, 2.51; *P* < 0.001). In addition, there was significant heterogeneity between studies (*I*^2^ = 96.1%, *P* < 0.001; Figure 8).

#### APACHE II

**Figure 9:**
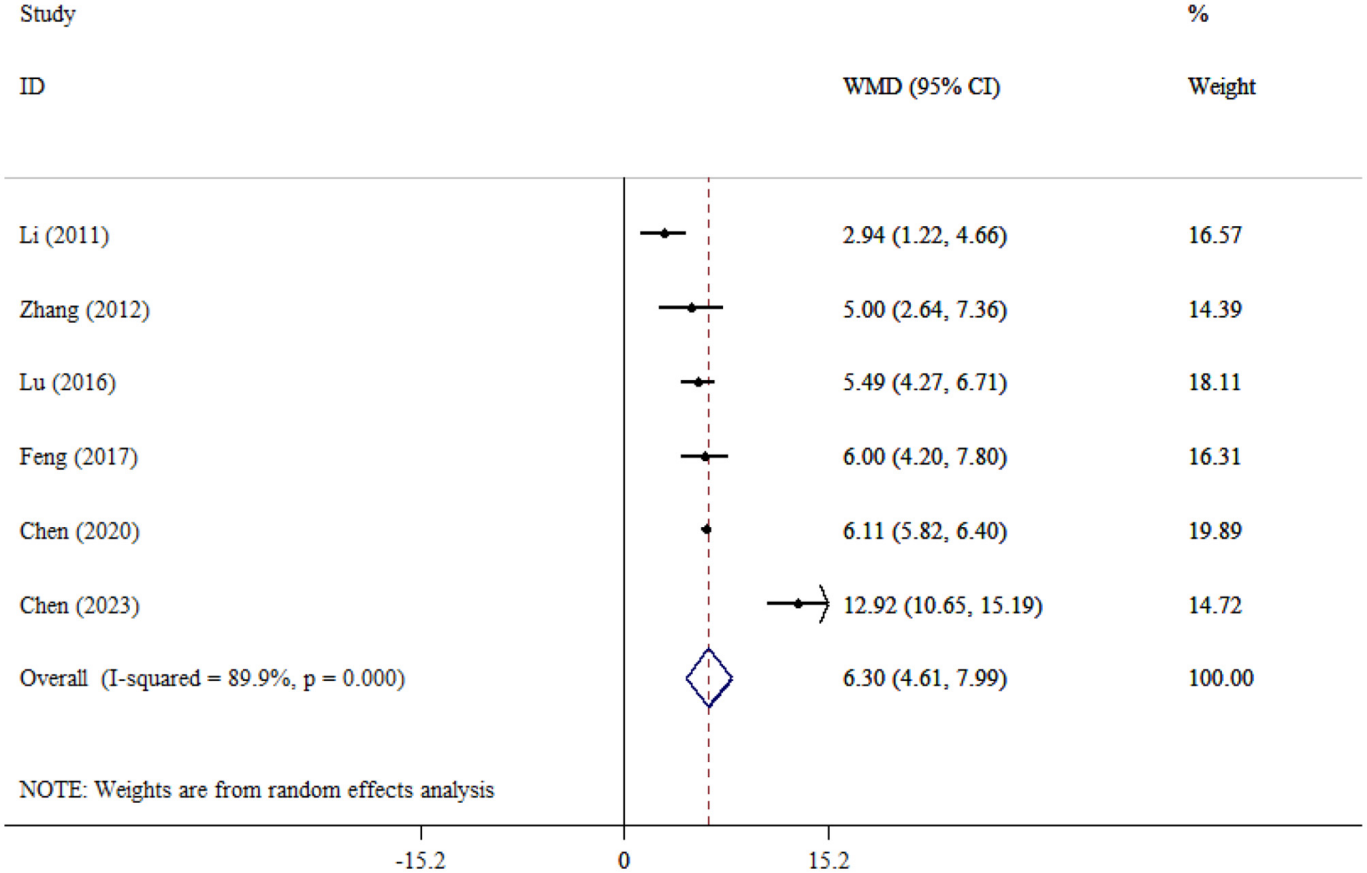
Forest plot evaluates the association between serum neurogenic biomarker levels and APACHI.

Six studies attempted to compare the APACHE II score between SAE and NE patients and demonstrated that APACHE II scores were significantly higher in the SAE group (WMD = 6.30; 95% CI: 4.61, 7.99; *P* < 0.001). Substantial heterogeneity was observed among studies (*I*^2^ = 99.7%, *P* < 0.001; Figure 9).

#### SOFA

**Figure 10:**
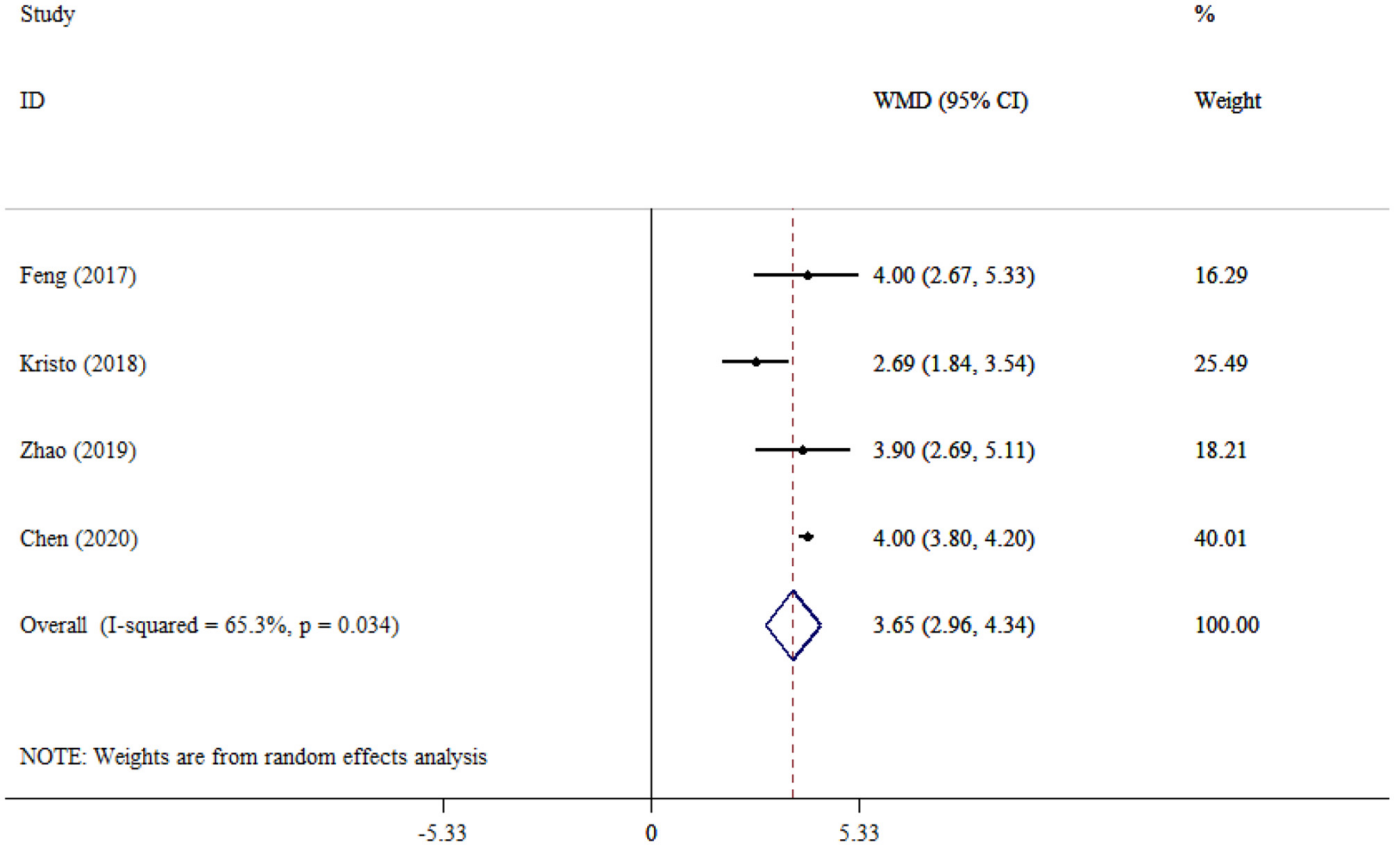
Forest plot evaluates the association between serum neurogenic biomarker levels and SOFA.

The pooled effect size of four studies evaluating the SOFA scores between SAE and NE patients showed that the SOFA scores were significantly increased in SAE compared to NE patients (WMD = 3.65; 95% CI: 2.96, 4.34; *P* < 0.001), pointing to greater organ dysfunction. In addition, moderate to high heterogeneity was observed among the studies (*I*^2^ = 65.3%, *P* = 0.034; Figure 10).

#### Death

**Figure 11:**
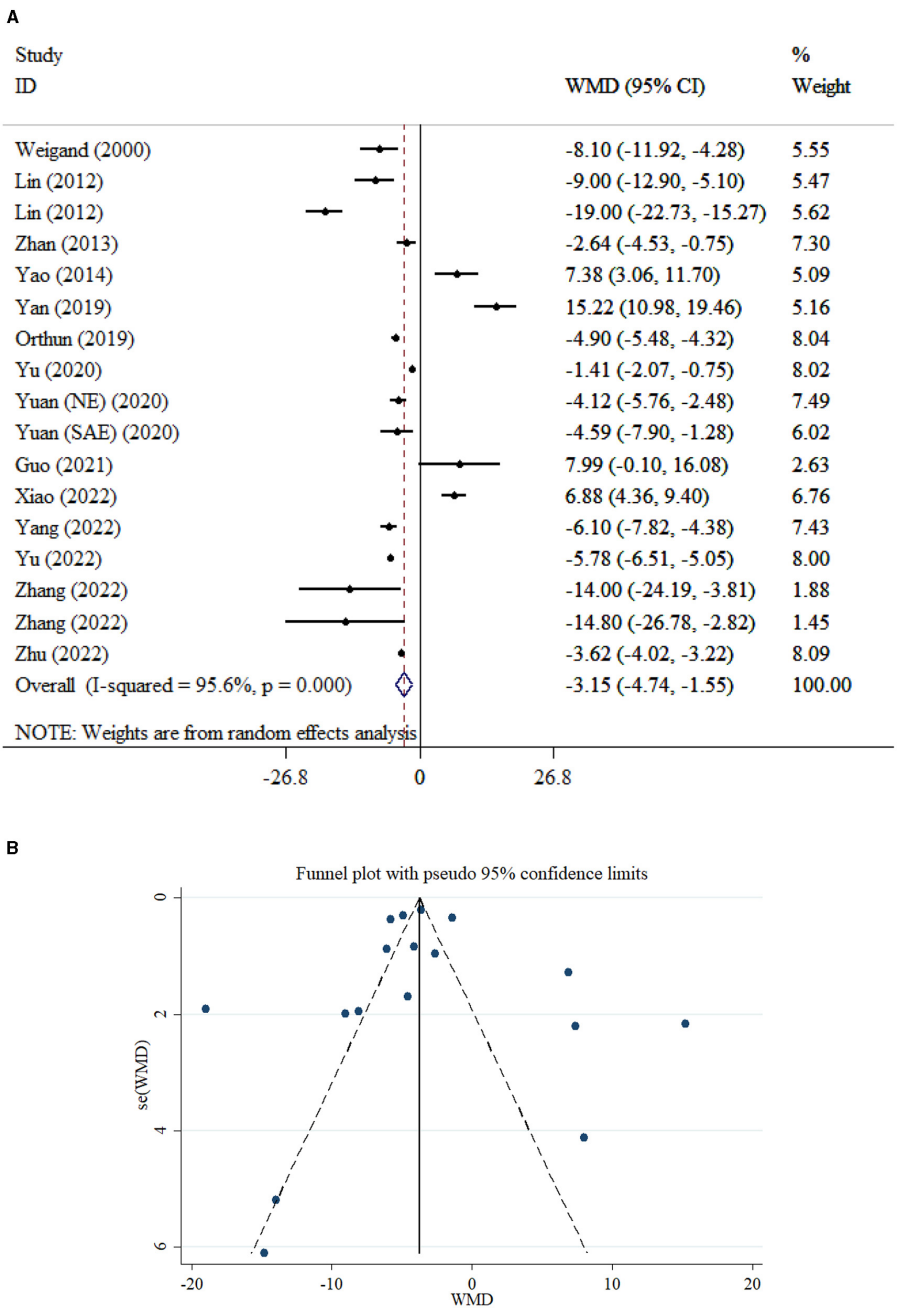
Forest plot **(A)** and funnel plot **(B)** evaluate the association between serum neurogenic biomarker levels and death in patients.

Pooled data from 17 studies (948 survival vs. 446 deaths) comparing mortality between patients with SAE and NE demonstrated significantly lower mortality in the NE group (WMD = −3.15; 95% CI: −4.74 to −1.55; *P* < 0.001). Substantial heterogeneity was observed across studies (*I*^2^ = 95.6%, *P* < 0.001; Figure 11A). Subgroup analysis indicated that, among NE patients, mortality was significantly lower in older adults (>55 years; *P* = 0.003), whereas no significant difference was observed in younger adults (*P* = 0.0963). In the NE group, mortality differences between older (*P* = 0.865) and younger (*P* = 0.233) adults were also non-significant. In addition, mortality was significantly lower when samples were collected after 1 day of sepsis onset in SAE patients (*P* < 0.001). While there was no significant difference in mortality when samples were collected within 1 day (*P* = 0.070; Table 2). No small-study effect was detected based on Egger's test (*P* = 0.290) and Begg's test (*P* = 0.650). Additionally, visual inspection of the funnel plot (Figure 11B) revealed an asymmetric distribution, suggesting potential publication bias.

#### Sensitivity analysis

In the sensitivity analysis, the removal of any single study did not affect the overall ES estimate for NSE, S100β, APACHE II, GFAP, TAU, UCH-L1, SOFA, and mortality.

## Discussion

SAE is an important complication of sepsis and requires critical management. In this regard, several neurogenic biomarkers and clinical severity scoring systems have been developed. To this end, this updated meta-analysis evaluated a panel of neurogenic biomarkers, including NSE, S100β, APACHE II, GFAP, TAU, UCH-L1, SOFA, and mortality, to differentiate between sepsis patients with and without encephalopathy. These biomarkers provide valuable insights into the underlying SAE pathophysiology and disease severity using the APACHE II and SOFA scoring systems. Moreover, mortality rates were also evaluated and compared between SAE and NE patients.

Accordingly, serum NSE levels were significantly elevated in SAE patients compared to NE patients. NSE, an enolase isoenzyme expressed in neurons, enters the bloodstream following neurogenic injury (11). An excessive increase in released NSE indicates neuroinflammation, which has important clinical implications. Despite the high heterogeneity (I^2^ = 98.9%), the increase in NSE was consistent across both age subgroups (<50 and >50 years) in SAE patients, suggesting that the neurogenic injury is an age-independent phenomenon. Similarly, despite high heterogeneity, elevated NSE levels were also observed in NE patients across both age subgroups (<50 and >50 years). This finding suggests that neurological dysfunction may occur even in the absence of sepsis. Furthermore, subgroup analysis demonstrated that elevated NSE levels can be observed regardless of the timing of sample collection (within 1 day or beyond 1 day after sepsis onset). These findings suggest that elevated NSE levels may reflect neuroinflammation during the acute phase and subsequent neurodegeneration during the prolonged phase. We also explored the pediatric population, though limited studies were available, and found that serum NSE levels were significantly higher in the SAE group than in the NE group, both in adults and children. Thus, there appears to be no age limitation for the diagnostic value of serum NSE in SAE. This finding is consistent with a previous study (15).

Pooled data showed that serum S100β levels were significantly higher in SAE patients compared to NE patients. S100β is predominantly expressed by astrocytes and is released into the peripheral circulation in response to neurogenic injury (44). Similarly, elevated serum levels of S100β have been widely recognized as a surrogate marker of blood–brain barrier (BBB) dysfunction, which plays an important role in the development of neuroinflammation (45). Thus, increased S100β levels may serve as a hallmark of BBB permeability. As indicated in the subgroup analysis based on age groups, S100β levels were significantly elevated in both younger (<55 years) and older (>55 years) adults, in both SAE and NE groups, despite the presence of substantial heterogeneity (*I*^2^ = 99.7%). This finding underscores that glial response to sepsis may occur in all age groups. However, Weigand et al. reported no significant difference in serum S100β between sepsis survivors and non-survivors (46). Although Glasgow Coma Scale (GCS) scores have been shown to be correlated with S100β levels in the diagnosis of SAE (23), additional diagnostic approaches and complementary methodologies are warranted to enhance diagnostic accuracy and support the development of treatment guidelines. In this regard, Cohen et al. introduced S100β as a marker for cognitive dysfunction in SAE too (47). Similarly, Calsavara et al. pointed to the possible association between serum S100β and anxiety and depression in SAE individuals (48). Previous studies have suggested a bidirectional association between S100β and SAE, indicating that elevated S100β levels may contribute to the development of SAE, while SAE itself may further elevate S100β levels through an as-yet unknown mechanism. Zhang et al. found that S100B may regulate mitochondrial dynamics through the RAGE/ceramide pathway, which results in cognitive dysfunction (49). Although each biomarker represents distinct mechanisms, the combined evaluation of NSE and S100β may provide insights into neuronal damage and blood–brain barrier disruption, which contribute to increased sensitivity and efficacy of SAE diagnosis.

However, the combined analysis of these studies illustrated increased levels of glial and neurogenic components, including GFAP, TAU, and UCH-L1, in SAE compared to NE individuals. The pronounced elevation of GFAP (WMD = 62.28; 95% CI: 45.42–79.14) points to a key role for astroglial activation and injury in SAE pathophysiology. This finding supports the possible use of GFAP as a threshold indicator for severe SAE, pending further studies to establish cut-off values. In addition, the consistent elevation of TAU and UCH-1 reveals axonal and neuronal degeneration involved in SAE pathophysiology.

Beyond neurogenic markers, clinical severity scores, including APACHE II and SOFA, were significantly higher in patients with SAE compared to NE individuals. These scoring systems are recognized for assessing the extent of physiological dysfunction in critically ill patients. The elevated scores observed in the SAE group suggest that encephalopathy may be linked to greater systemic severity, which may be driven by an increased inflammatory condition or multi-organ failure. Encephalopathy indicates a poor prognosis for patients suffering from sepsis (50). The elevated levels of neurogenic biomarkers alongside clinical severity indices such as APACHE II and SOFA highlight their role in the disease burden.

Overall, significant differences were observed between SAE and NE patients in studied outcomes, including NSE, S100β, and a particularly larger effect size for GFAP, underscoring the evaluation of the multi-biomarker panel approach. Future studies should investigate whether the combined assessment of NSE + S100β + GFAP can improve the sensitivity and specificity of SAE diagnosis compared with single markers specifically. Similarly, well-designed prospective studies are warranted to address this study gap and to validate the clinical utility of combined biomarker assessment in SAE. Accordingly, such studies will help translate biomarker-based panels into clinically applicable diagnostic tools.

In addition, the mortality rate was significantly lower in NE vs. SAE. The high heterogeneity across studies may trigger controversy over the results. As subgroup analysis provided further insights into age-related differences, mortality was significantly lower in older adults (>55 years) than among SAE patients. Age-specific physiological conditions and earlier recognition and treatment in older individuals may be responsible for these differences in the results. Whereas, mortality did not differ significantly across age groups in NE patients. Moreover, the mortality rate was lower in SAE individuals when sample collection was carried out more than 24 h after sepsis onset.

One of the strengths of this study is its evaluation of a broader range of neurogenic and severity-scoring biomarkers, offering a more comprehensive approach. However, this study has some limitations too. First, the diagnostic criteria for sepsis 1.0 have high sensitivity with low specificity. Second, there was considerable variability in the diagnostic criteria for SAE, which may undermine the robustness of comparisons and contribute to observed heterogeneity. It is recommended to use a standardized diagnostic marker. Third, this study has potential publication bias. Fourth, various assay methods, sample processing techniques, and timing of sampling may introduce variability in the results and deserve further elaboration in future research. Fifth, the limited number of included studies and their small sample sizes restrict the generalizability of findings, particularly for UCH-L1, SOFA, and APACHE II scores, as well as NSE outcomes in pediatric populations.

## Conclusion

This study highlights promising pharmacological targets for preventing SAE. In total, increased levels of common serum neurogenic biomarkers and mortality were associated with SAE, which may be useful in the diagnosis of SAE patients. The findings for UCH-L1, SOFA, and APACHE II scores should be interpreted cautiously, as they preliminarily suggest potential value but require further validation in larger, well-designed studies.

## Data Availability

The datasets presented in this study can be found in online repositories. The names of the repository/repositories and accession number(s) can be found in the article/supplementary material.
